# Efficient iodine capture from water using a functionalized covalent organic framework

**DOI:** 10.1039/d5ra09537a

**Published:** 2026-02-19

**Authors:** Mahsa Jahantigh, Mostafa Khajeh, Ali Reza Oveisi, Saba Daliran, Mansoureh Rakhshanipour

**Affiliations:** a Department of Chemistry, Faculty of Sciences, University of Zabol P. O. Box: 98615-538 Zabol Iran m_khajeh@uoz.ac.ir; b Department of Organic Chemistry, Faculty of Chemistry, Lorestan University Khorramabad 68151-44316 Iran oveisi.a@lu.ac.ir +98-543-2226765

## Abstract

The development of effective iodine adsorbents is a vital and challenging goal in radioactive waste management. In this study, we functionalized a covalent organic framework (COF-366) with amidoxime groups (AO) to create a new porous organic polymer (POP-AO), designed specifically for iodine capture. We systematically examined several key factors, including contact time, pH, initial iodine concentration, and the effect of competing ions on adsorption performance. Notably, POP-AO achieved an impressive iodine uptake of 909.1 mg g^−1^, exceeding many previously reported materials. This high capacity mainly results from Lewis acid–base interactions between the electron-rich AO groups and electrophilic iodine. Kinetic studies indicated that the adsorption follows a pseudo-second-order model. Moreover, the material maintained its high capacity of 909.1 mg g^−1^ after seven regeneration cycles, demonstrating excellent reusability. This research not only offers a promising strategy for radioactive iodine remediation but also establishes POP-AO as a durable and effective adsorbent for practical use.

## Abstract 2

An amidoxime-functionalized COF-366 enables ultrafast, high-capacity molecular iodine capture from water (909.1 mg g^−1^), driven by charge-transfer interactions, with excellent reusability over seven cycles.

## Graphical abstract

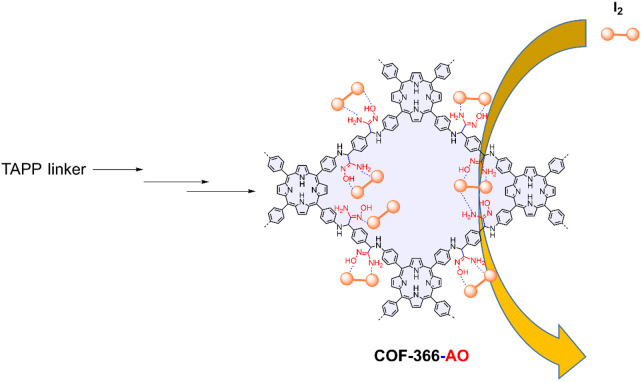

## 1. Introduction

The safe and effective management of iodine, especially its radioactive isotopes, is a critical global challenge. Radioactive iodine-129, with a half-life of 15.7 million years, poses a long-term environmental and health threat, as seen in nuclear incidents such as Fukushima.^1–3^ Additionally, iodine-related concerns extend to water treatment, where disinfection processes can create highly toxic iodinated by-products.^4–6^ The health implications are severe since the thyroid gland concentrates iodine; exposure to radioactive isotopes can cause cell damage and increase the risk of cancer.^7^ Consequently, developing advanced materials for efficient iodine capture is essential for environmental protection and public health.

Adsorbents such as activated carbon^8^ and zeolite^9^ have been used for iodine capture but often face limitations like low capacity and poor reusability. The field has progressed to the development of more sophisticated porous materials, including metal–organic frameworks (MOFs), porous organic polymers (POPs), and silver-impregnated adsorbents. Each class presents distinct advantages and trade-offs. For instance, silver-based materials exhibit strong chemisorption for iodine but are costly and can suffer from silver leaching.^10^ MOFs, like MIL-101(Cr),^11^ offer high surface areas and tunability, yet reported iodine capacities can be modest and their hydrothermal/chemical stability is not guaranteed. While novel materials such as graphene aerogels,^12^ porous aromatic frameworks,^13^ and microporous polymers^14^ exhibit improved performance, they face challenges owing to their random pore structures and/or insufficient functionalization for selective capture. An ideal adsorbent would have a high density of specific binding sites, a well-ordered porous structure, and stability under the conditions of use, thereby enhancing capacity, selectivity, and kinetics.

Covalent organic frameworks (COFs) are a subclass of porous organic polymers (POPs) made up of covalently linked organic building blocks, making them a promising class of materials that can meet these requirements.^15–17^ Their crystalline and porous structures, along with high surface areas and intrinsic design, allow for precise control over their properties. They can be engineered into composite formats (*e.g.*, COF@cotton)^18,19^ to improve practicality, yet the direct integration of optimal functionality within a robust scaffold remains a key challenge. A significant advantage of COFs is their ability to undergo post-synthetic modification (PSM) to introduce sophisticated functionalities that are challenging to incorporate during *de novo* synthesis.^20^ This strategy enables the tailoring of COFs for specific applications, including pollutant capture.^21,22^

For iodine adsorption, the amidoxime functional group is appealing because it contains both an oxime and an amino group linked to a carbon atom, offering coordinating sites for robust binding.^23–25^ The effectiveness of amidoxime has been demonstrated in polymers; for example, amidoxime-grafted polyacrylonitrile achieved an impressive iodine capacity of 1534.4 mg g^−1^.^24^ However, integrating this powerful functional group into a highly ordered porous COF scaffold could potentially create a superior adsorbent that enables rapid capture under ambient conditions, addressing the limitations of slower kinetics in some MOFs or the lower intrinsic capacity of unmodified COF composites.

**Scheme 1:**
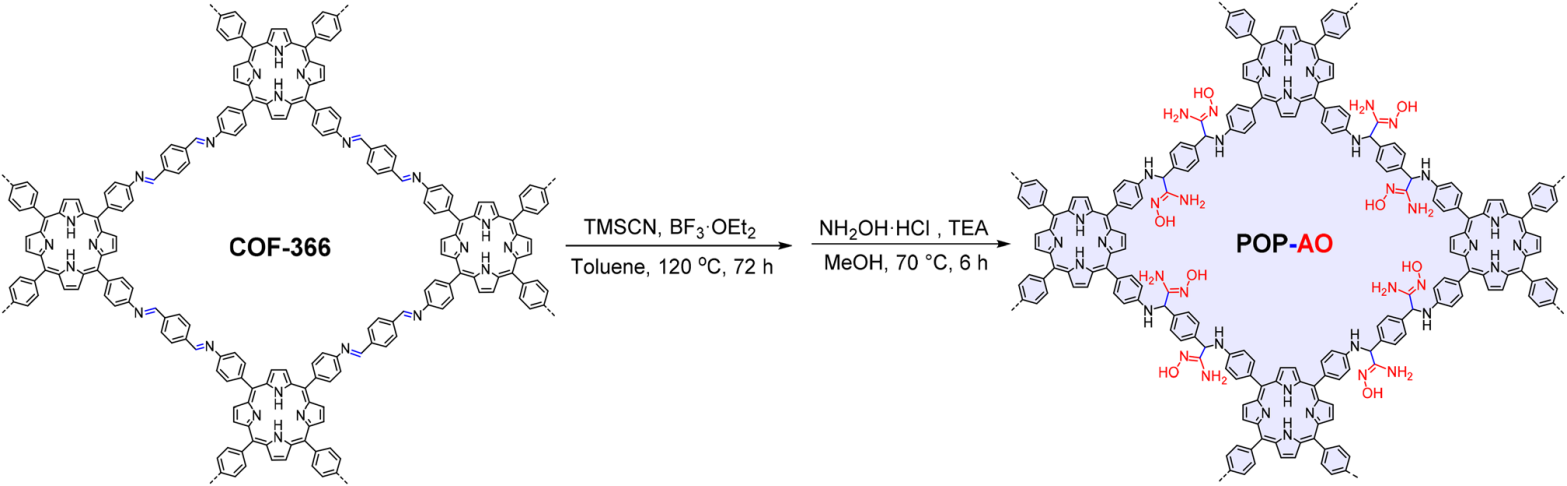
Synthetic strategy for the preparation of POP-AO.

In this work, we report the design and synthesis of an amidoxime-functionalized swelling POP for highly efficient iodine capture. We first synthesized a porphyrin-based COF-366 from tetrakis(4-aminophenyl)porphyrin (TAPP) and terephthalaldehyde.^26^ This framework was then transformed through a two-step PSM process: initial cyanation with trimethylsilyl cyanide (TMSCN) using a validated procedure for COFs,^27^ followed by conversion of the nitrile groups to amidoxime using hydroxylamine.^20,23,24,28^ The PSM led to a loss of long-range structural order, as confirmed by powder X-ray diffraction, a feature typical of COFs.^29^ Consequently, the final product is more accurately classified as an amorphous POP, designated as POP-AO. Despite this structural transition from a crystalline framework, the material retained its porous nature and was successfully endowed with a high density of amidoxime functionalities. We therefore systematically evaluated the iodine adsorption performance of POP-AO. Key parameters investigated included contact time, solution pH, the influence of competing ions, and reusability over multiple adsorption–desorption cycles (Scheme 1).

## 2. Materials and method

### 2.1. Materials

All the following materials and solvents including acetone, methane, ethanol, acetic acid, pyridine, ammonia, HCl, propionic acid, *ortho*-dichlorobenzene, trimethylamine (TEA), *n*-butanol, hydroxylammonium chloride (NH_2_OH·HCl), pyrrole, trimethylsilyl cyanide (TMSCN), boron trifluoride etherate (BF_3_·OEt_2_), 4-nitrobenzaldehyde, SnCl_2_ and terephthalaldehyde were obtained from Merck or Sigma-Aldrich (Germany, KGaA) analytical grade and employed directly without any additional processing. HCl and NaOH (0.1 mol L^−1^) were used to maintain a constant pH throughout the test. Tetrakis(4-aminophenyl)porphyrin (TAPP)^30^ and COF-366 (ref. 26 and 31) were synthesized following previously reported procedures. Absolute ethanol (≥99.5%, analytical grade) was obtained from Merck (Germany) and used as received for the preparation of iodine stock solutions. Detailed information about all the instruments used in this work, as well as their specifications, is provided in the SI.

### 2.2. Synthesis of POP-AO

A mixture of COF-366 (4 mg), BF_3_·OEt_2_ (10 µL), and TMSCN (15 µL) in anhydrous toluene (2 mL) was added to a 10 mL penicillin vial. The vial was sealed with a rubber septum and an aluminum crimp cap, then evacuated and purged with nitrogen three times to establish an inert atmosphere. The reaction mixture was heated at 120 °C for 72 h in an oil bath on a digital hotplate with magnetic stirring. After cooling to room temperature, the product was sequentially washed with tetrahydrofuran and dichloromethane (three times each) *via* centrifugation. The resulting solid was dried to yield the cyano-functionalized intermediate (cyanation step). The cyano-functionalized intermediate (100 mg) was suspended in anhydrous methanol (4 mL) in a 10 mL vial. NH_2_OH·HCl (41 mg) and TEA (89 µL) were added, and the vial was sealed. The reaction mixture was stirred magnetically at 70 °C for 3 h. Subsequently, additional NH_2_OH·HCl (20 mg) and TEA (44 µL) were added, and heating was continued at 70 °C for a further 3 h. The final product was isolated by centrifugation, washed with deionized water and ethanol (three times each), and dried under vacuum in a stepwise manner (50 °C for 2 h, then 90 °C for 8 h) to yield POP-AO. The cyano-functionalization and its subsequent conversion to amidoxime follow well-established methodologies.^20,23,24,27,28^

### 2.3. Solid-phase extraction procedure

A dispersive solid-phase procedure was used to investigate iodine extraction by the POP-AO adsorbent and the possible mechanism. We prepared a standard solution of molecular iodine with an initial concentration of 0.05 mol L^−1^ in 100 mL of water and subsequently diluted to obtain various concentrations. We immersed 5 mg of POP-AO in 25 mL of iodine solution at multiple concentrations. A stock solution of molecular iodine (0.05 mol L^−1^) was prepared by dissolving iodine in 10.0 mL of ethanol, by gradually diluting to 100.0 mL with deionized water under continuous ultrasonication. Working solutions at various concentrations were prepared by suitable dilution of the stock solution. After that, the solution was sonicated for the chosen time, and the extraction was done. The solution was centrifuged for 5.0 min at 5000 rpm, and the supernatant was collected. The concentrations of iodine solution at various time intervals were measured at 458 nm wavelength^1^ in the supernatant using a UV-Vis spectrophotometer. The equilibrium adsorption capacity (*q*_e_, mg g^−1^) of the POP-AO was estimated by the equation:

1 *q*_e_ = (*C*_0_ − *C*_e_)*V*/*m*

where *C*_0_ and *C*_e_ (mg L^−1^) represent concentrations of iodine in solution initially and after adsorption, respectively. *m* (g) represents the mass of the POP-AO, and *V* (L) is the volume of the solution.

To investigate the adsorption mechanisms and investigate adsorption behavior, kinetic models such as pseudo-first-order and pseudo-second-order, and isotherm models such as Langmuir and Freundlich were used. For the kinetic study, each sample was subjected to nine experiments. The iodine concentration was determined after 1, 2, 3, 5, 7, 10, 15, 20, and 25 minutes. The optimum conditions for iodine extraction were determined: sample volume of 25 mL, mass of adsorbent of 5.0 mg, solution pH of 6.0, and extraction time of 20 min.

## 3. Results and discussion

### 3.1. Characterization

FT-IR spectroscopic analysis revealed significant structural modifications upon cyanide functionalization of the COF framework (Fig. S1). The emergence of an absorption band at 2217 cm^−1^ confirmed successful cyanide group incorporation, while a broad band at 3419 cm^−1^ indicated N–H stretching vibrations from amine groups. Subsequent conversion to the amidoxime derivative (POP-AO) through NH_2_OH·HCl treatment resulted in substantial attenuation of the –C≡N peak, demonstrating effective nitrile group conversion.^24^ The appearance of new characteristic bands at 1174 cm^−1^ (C–N stretch) and 1621 cm^−1^ (C=N stretch of the porphyrin linker), along with a broad N–H/O–H stretching band at 3450 cm^−1^, collectively confirmed the formation of POP-AO.^23^ These distinct spectral transformations provide conclusive evidence of successful amidoxime (–C(=NOH)NH_2_) functionalization.

**Fig. 1:**
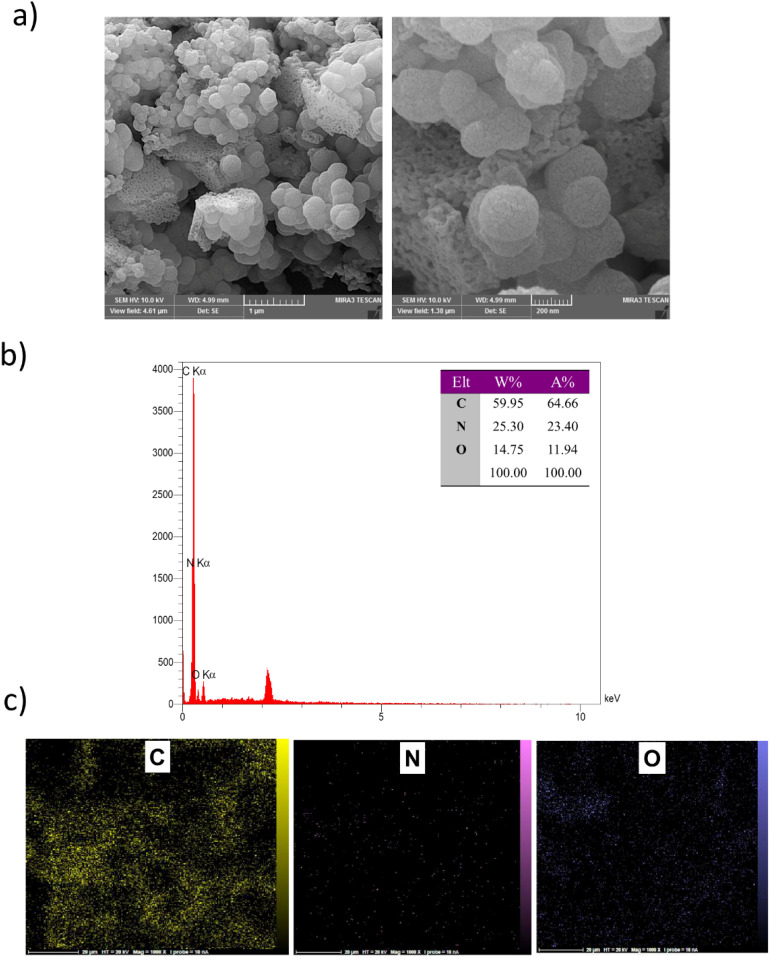
(a) SEM image; (b) EDX spectrum with elemental composition (wt% and at%); and (c) EDX elemental mapping of POP-AO.

SEM images of POP-AO reveal aggregated spherical particles with a size distribution of 50–300 nm (Fig. 1a). In contrast, the morphology of the solid appears unchanged after modification, as seen in a comparison of the pristine (Fig. S2) and modified materials. EDX analysis (Fig. 1b) confirms the presence of nitrogen (N), carbon (C), and oxygen (O), verifying the successful incorporation of amidoxime functional groups into the COF structure. The oxygen content (11.94 wt%) primarily originates from the amidoxime (AO) groups introduced during PSM. The elemental composition (at%) is 64.66% (C), 23.40% (N), and 11.94% (O), with corresponding weight percentages (wt%) of 59.95% (C), 25.30% (N), and 14.75% (O).

Elemental mapping (Fig. 1c) shows a consistent distribution of C, N, and O throughout the POP-AO structure, confirming even functionalization without phase separation or elemental clustering. These results indicate a well-synthesized and evenly modified material.

**Fig. 2:**
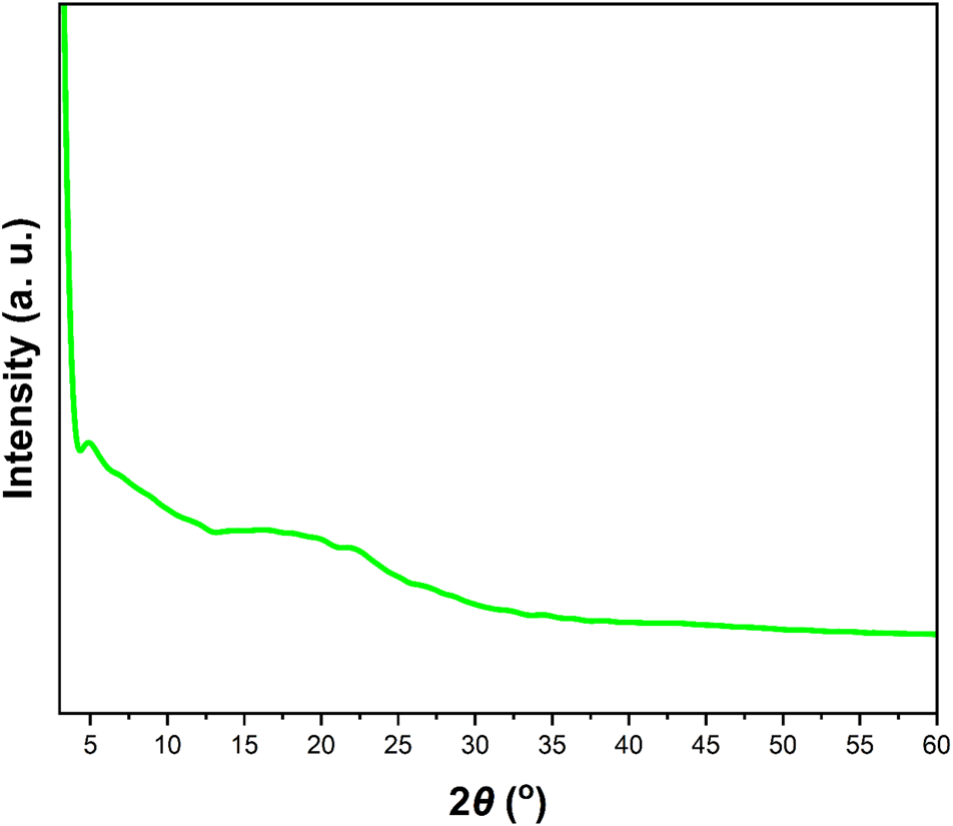
PXRD pattern of POP-AO.

The X-ray diffraction pattern (XRD) indicates that the POP-AO lacks long-range crystalline order, exhibiting a semi-amorphous structure (Fig. 2), consistent with prior observations in post-synthetically modified imine-based COFs.^29,31^ This is evident from the sharper diffraction pattern of the pristine COF (Fig. S3).

The textural properties of the material, as confirmed by nitrogen physisorption (Fig. S4), is a critical but significantly altered factor in its performance. The N_2_ adsorption–desorption isotherm exhibits a hybrid shape, combining features of Type III and Type IV isotherms. The convex curvature at low-to-medium relative pressures indicates weak initial gas–solid interactions and a pronounced loss of microporosity compared to the pristine COF-366. At high relative pressure (*P*/*P*^0^ > 0.8), a sharp rise accompanied by a distinct hysteresis loop emerges, confirming the presence of a well-defined mesoporous network through capillary condensation. The corresponding pore size distribution (inset, Fig. S4) confirms the material is mesoporous, with pore widths concentrated in the 2.5–5 nm range. While this architecture facilitates mass transport, the measured BET surface area of 65 m^2^ g^−1^ and pore volume of 0.117 cm^3^ g^−1^ are markedly lower than values typical for pristine COF-366 (often >1000 m^2^ g^−1^). This substantial reduction quantifies a significant loss of porosity upon PSM, indicating pore blockage or partial collapse during the functionalization process. Despite this reduction, the retained functionalized swelling polymer, combined with the high density of newly installed amidoxime functionalities, is the key factor behind the enhanced iodine adsorption capacity demonstrated in this study.

The thermal stability of POP-AO was assessed by thermogravimetric analysis (TGA) under air. The profile shows a significant mass loss step with an onset temperature of 420 °C (Fig. S5), which we attribute to the decomposition of the functionalized organic framework. This onset temperature indicates that POP-AO retains high thermal robustness after post-synthetic modification. In comparison with pristine porphyrin-based COF-366, which is generally reported to exhibit decomposition onset temperatures above 450 °C, the slightly lower onset observed for POP-AO is reasonable and can be attributed to the partial loss of long-range crystallinity and the introduction of amidoxime functionalities during post-synthetic modification. Importantly, POP-AO remains thermally stable well above typical operating and regeneration temperatures, indicating that the post-synthetic modification does not compromise its practical thermal stability.

### 3.2. Batch adsorption experiments

To determine the impact of amidoxime functionalization on iodine adsorption, we examined how POP-AO compared to COF-366 (pristine) when exposed to iodine under identical experimental conditions (pH 5.0, 1.0 g per L iodine concentration, 20 min contact time, 5.0 mg adsorbent, 25.0 mL sample volume). While COF-366 (pristine) adsorbed 489 mg per g iodine, POP-AO adsorbed 563 mg per g iodine within these same experimental conditions, demonstrating a 15.1% increase in iodine adsorption capacity of POP-AO compared to COF-366 (pristine). This comparison indicates that the presence of amidoxime groups will be instrumental in achieving enhanced iodine adsorption performance. Even though COF-366 has a well-defined crystalline structure and has the potential for a high surface area, without chemical functionality for enhanced binding capacities, COF-366 (pristine) will not efficiently adsorb iodine. The information presented indicates that the enhancement of iodine adsorption *via* chemical modifications to POP-AO is far greater than would be expected based solely on the differences in surface area or crystallinity between the two materials. The amidoxime groups serve as specific binding sites for the formation of stable charge transfer complexes between iodine and the amidoxime through Lewis acid/base interactions.

**Fig. 3:**
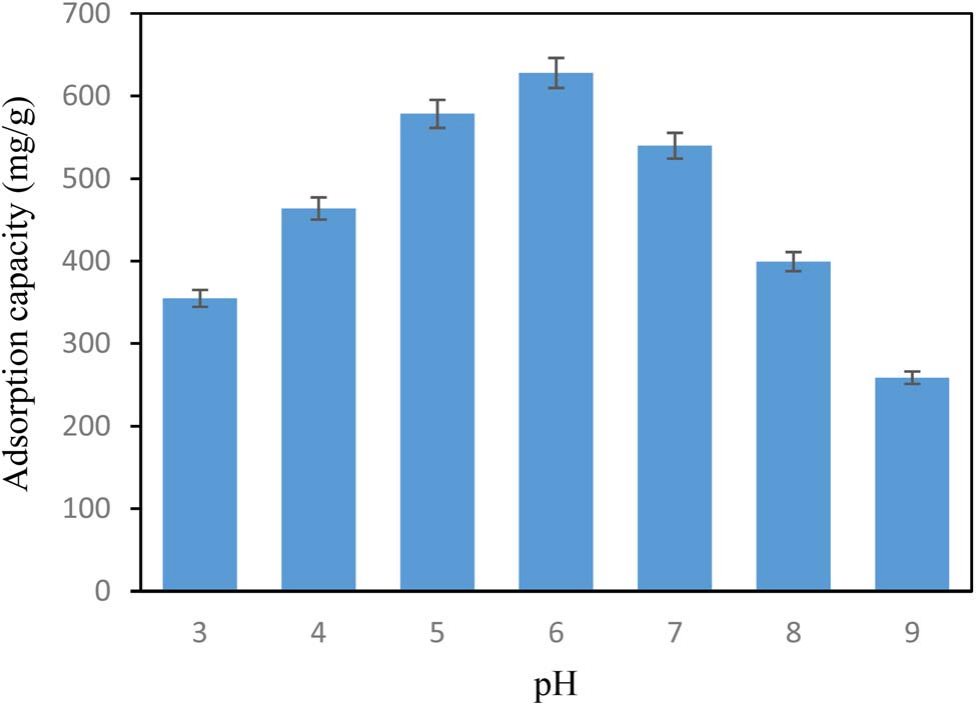
The iodine adsorption capacity in different pH (iodine concentration, 1.0 g L^−1^; time, 20.0 min; adsorbent mass, 5.0 mg; sample volume, 25.0 mL, RSD% = 2.9).

The pH of the solution plays an essential role in determining iodine and the surface charge properties of POP-AO. To determine the impact of pH on the iodine adsorption capacity, experiments were performed under various pH conditions ranging from 3 to 9 (Fig. 3). From the results, it was indicated that with a pH of 6, after a period of 20.0 min, the maximum adsorption capacity of POP-AO was 627.75 mg g^−1^. However, the capacities were lower with a pH range of 3, 4, and 5, with a capacity of 354.65, 463.81, and 578.76 mg g^−1^, respectively. With a pH range of 7, 8, and 9, the capacity was lower with a value of 540.0, 399.24, and 258.92 mg g^−1^, respectively. pH value of the solution is one of the most important factors in determining the speciation of iodine and the surface charge character of POP-AO. Above pH 10, molecular iodine is destabilized and undergoes a transformation to produce iodate (IO_3_^−^) and iodide (I^−^),^1^ making it colorless and non-absorbent. Below pH 7, molecular I_2_ is most preferentially adsorbed on electron-rich nitrogen and oxygen atoms of the amidoxime moiety *via* Lewis acid–base interactions. Below pH 6, in an acidic environment, protonation of the amidoxime functionality (–C(=NOH)NH_2_ to –C(=NOH_2_^+^)NH_3_^+^) will reduce electron density, making it less efficient in adsorption. However, above pH 6, molecular I_2_ would gradually disproportionate to I^−^ and IO_3_^−^, which would be less efficient in interaction with the neutral amidoxime functionality than molecular I_2_. Thus, the optimal pH of 6 is established to ensure the stability of molecular I_2_ and retain the electron-donating nature of the amidoxime functionality.

**Fig. 4:**
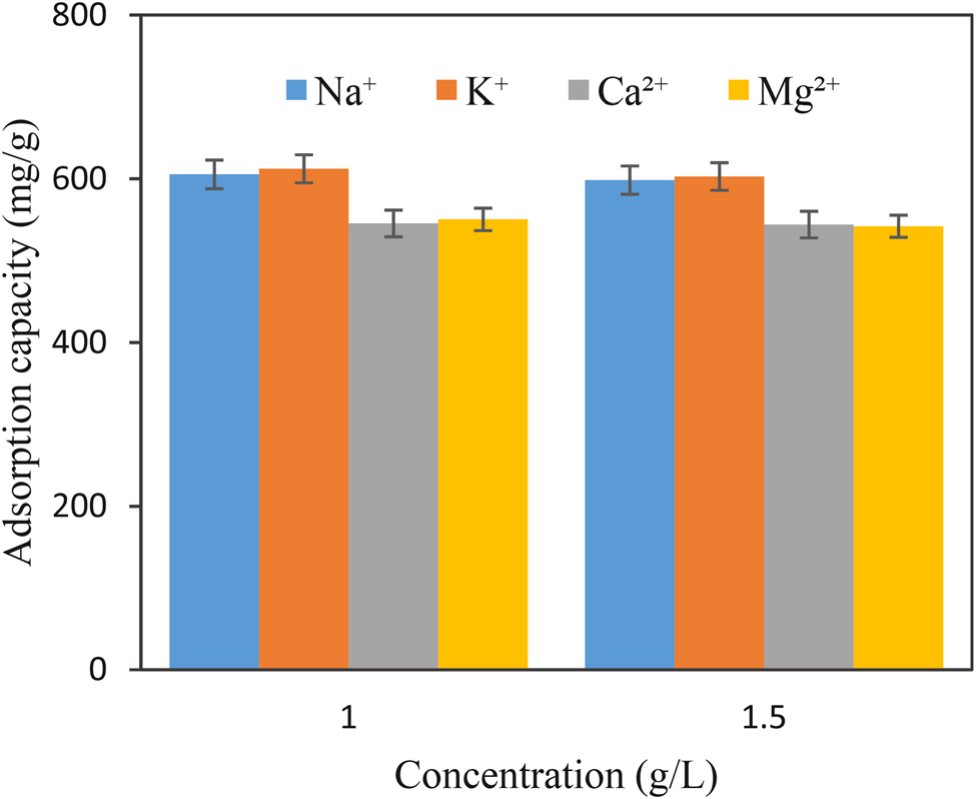
The effect of the coexistence of ion concentration on the adsorption capacity of iodine (iodine concentration, 1.0 g L^−1^; time, 20.0 min; adsorbent mass, 5.0 mg; sample volume, 25.0 mL, RSD% = 2.5 to 3.0).

Different ions' presence carries out a key role in shaping the adsorption dynamics by enhancing ionic strength and creating competition for adsorption sites. To explore these impacts, a range of experiments was meticulously designed with 1.0 and 1.5 g L^−1^ concentrations of electrolytes. As the concentrations of the three metal cations increased, the adsorption capacity of POP-AO did not significantly change (Fig. 4). This demonstrated the excellent anti-ions interference capability of POP-AO as well as its application potential in the extraction of iodine from seawater.

The reduced adsorption capacity for the divalent cations (Ca^2+^ and Mg^2+^) relative to the monovalent cations (Na^+^ and K^+^) for POP-AO materials could be ascribed to the stronger influence of ionic strength and very weak competition for the adsorption site. The divalent cations have a higher impact on the ionic strength of the solution, causing the electrical double-layer region to collapse and resulting in a slight agglomeration of the adsorbent materials and a slight reduction in accessibility to the adsorption site. The other factor is the weaker coordination interactions compared to the strong I_2_–amidoxime interaction for nitrogen or oxygen atoms on the amidoxime functionality for the divalent cations. Nevertheless, the differences are very trivial (<10%), ensuring high selectivity for iodine and the satisfactory efficiency of POP-AO materials for industrial applications even in higher-salinity water such as seawater.

To further assess the selectivity of POP-AO, the effect of common anionic interferents was studied under optimized conditions, where the concentration of I_2_ was 0.5 g L^−1^ and with anion concentrations at 3-fold excess (1.5 g L^−1^). The iodine recovery in the presence of Cl^−^, Br^−^, NO_3_^−^, SO_4_^2−^, PO_4_^3−^, and CO_3_^2−^ were 94.8%, 93.8%, 93.6%, 91.4%, 89.4%, and 91.2%, respectively. These results reveal that common anionic interferents show minimal interference with iodine adsorption, confirming the excellent selectivity of POP-AO.

To identify the ideal contact time, adsorption experiments were performed at 1, 2, 3, 5, 7, 10, 15, 20, and 25 minutes of time (Fig. S7). The findings showed that iodine was adsorbed more rapidly during the first 15 minutes and reached equilibrium around 20 minutes, with minimal change observed during the 25-minute time point. This rapid establishment of equilibrium suggests that the iodine molecule has efficient accessibility to bind to the amidoxime sites in POP-AO. Kinetic experiments were used to investigate the desorption rate of iodine from aqueous samples and identify the adsorption process mechanisms. Herein, the iodine adsorption kinetics over POP-AO were studied using pseudo-first-order and pseudo-second-order models whose equations are presented below.^1^

2 Pseudo-first-order: ln(*q*_e_ − *q*_*t*_) = ln *q*_e,c_ − *k*_1_*t*

**Fig. 5:**
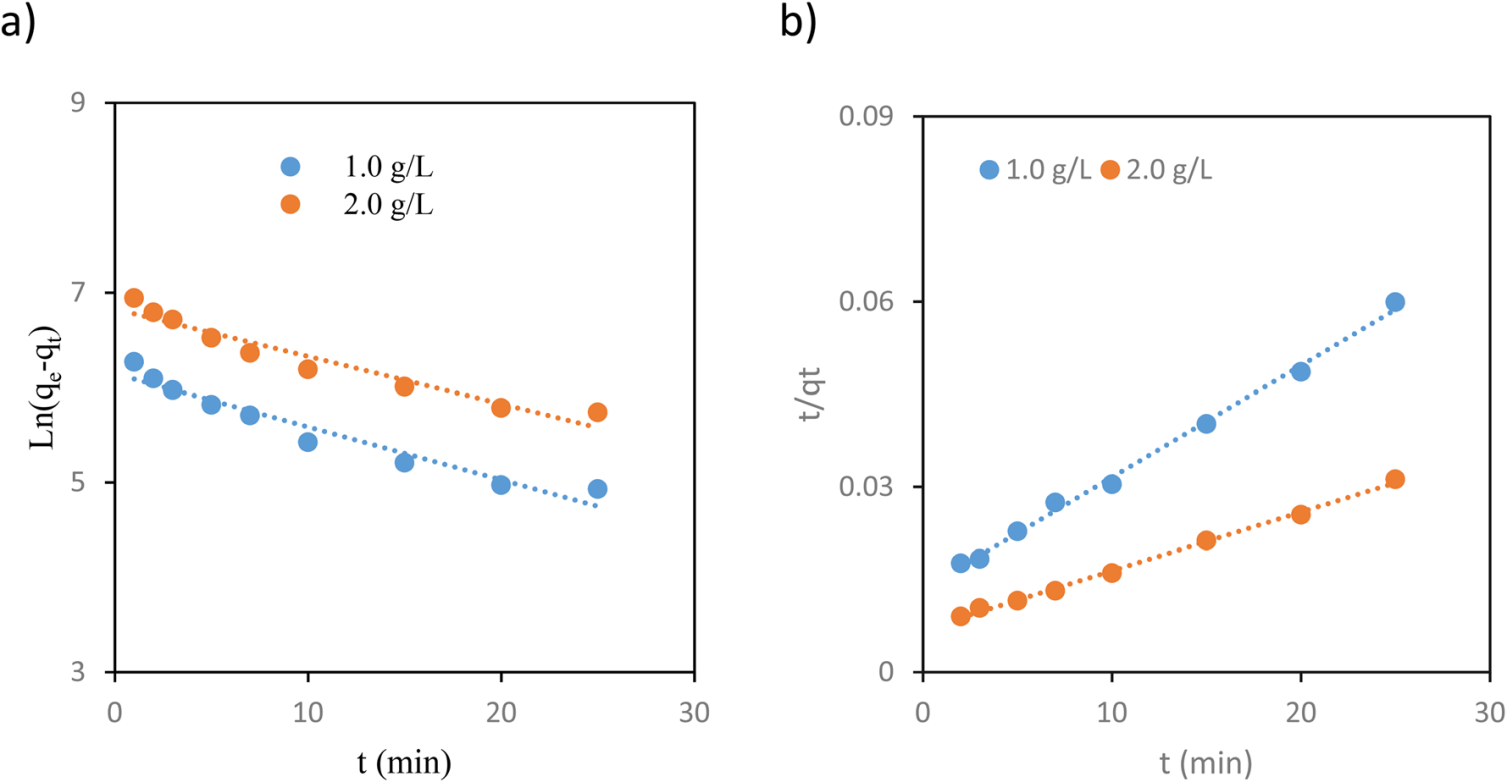
Plots of pseudo-first-order (a) and pseudo-second-order (b) kinetics for the adsorption of iodine on the POP-AO.

3
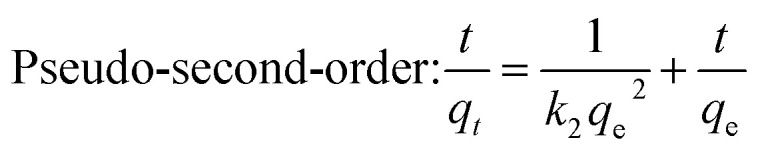
In this context, *q*_e_ and *q*_*t*_ (mg g^−1^) represent the iodine adsorption capacity of POP-AO at equilibrium and at a time *t* (min), respectively. The rate constant *k* and the equilibrium adsorption capacity (*q*_e_) were determined from the slope and intercept of the corresponding linear plots (Fig. 5) and are summarized in Table 1. The pseudo-first-order model indicates a physical adsorption mechanism, whereas the pseudo-second-order model describes a chemical adsorption process.^1^ The calculated parameters and determination coefficients (*R*^2^) for both models are presented in Table 1. Notably, based on the pseudo-second-order model, we found a higher determination coefficient (>0.990) and an adsorption capacity that closely matched the test results. The pseudo-second-order model appears more suitable for characterizing the adsorption kinetics of iodine onto POP-AO in aqueous solutions. According to the pseudo-second-order model of POP-AO, the kinetic rate constants (*K*_2_) were calculated at varying iodine concentrations. At 1.0 and 2.0 g L^−1^ iodine aqueous solutions, the constants were 1.49 × 10^−4^ and 8.57 × 10^−5^ mg g^−1^ h^−1^, respectively. The results suggest that the value of the adsorption rate constant (*K*_2_) reduces with an increase in iodine concentration as a result of increased competition for adsorption sites on the surface for chemisorption.^1^

**Table 1:** Kinetic parameters for iodine adsorption on POP-AO at various concentrations

Samples	Pseudo-first-order kinetic model			Pseudo-second-order kinetic model		
	*q*_e1_ (mg g^−1^)	*k*_1_ (h^−1^)	*R* ^2^	*q*_e2_ (mg g^−1^)	*k*_2_ (g mg^−1^ h^−1^)	*R* ^2^
1.0 g L	466.1	0.0559	0.941	555.6	2.4 × 10^−4^	0.996
2.0 g L^−1^	922.0	0.05	0.935	1111.2	11.7 × 10^−5^	0.997

Adsorption rates are determined by the rate-limiting step for porous materials, which is an essential factor to consider.^32^ Typically, the initial step in adsorption involves liquid film diffusion or boundary layer diffusion, where iodine transitions from the water sample to the surface of the POP-AO. Following this, intraparticle diffusion allows the adsorbate to enter the POP-AO pores. Finally, the adsorbate iodine less adheres to active sites on the surface through physical and chemical interactions.^33^ Either boundary layer diffusion, intraparticle diffusion, or a combination of both often controls the process of adsorbate molecule transfer. To assess the contribution of intraparticle diffusion, the Weber–Morris equation,^1^ as follows, is typically used to analyze this behavior.

4 *q*_*t*_ = *k*_ID_*t*^1/2^ + *C*

**Fig. 6:**
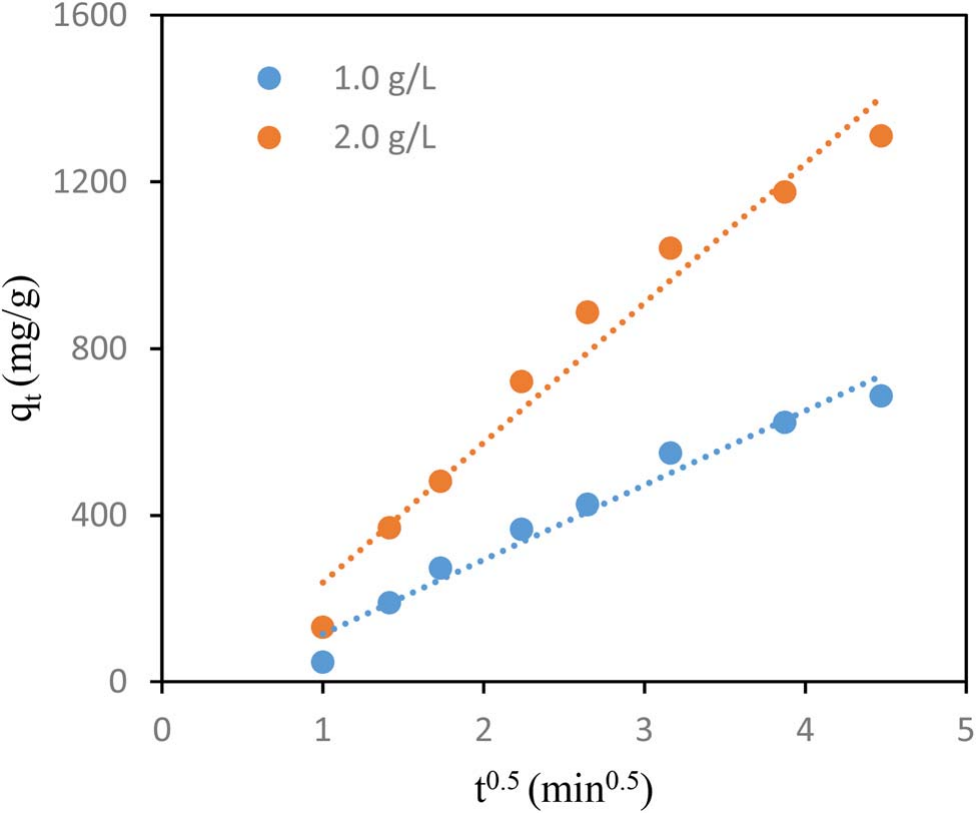
Intra-particle diffusion model plot for the adsorption of iodine on the POP-AO.

As empirical studies observe, the relationship between adsorption and time (*t*_1/2_) is typically linear.^34^ A linear regression plot indicates that the adsorption mechanism follows the intraparticle diffusion. When a plot intersects the origin, it suggests that intraparticle diffusion is a rate-limiting step. Conversely, if the plot deviates from the origin, it suggests the involvement of additional mechanisms in governing the adsorption process.^35^ For all experimental conditions, straight lines that did not cross the origin provided the best fit for regression plots (Fig. 6). This finding implies that intraparticle diffusion was not the only rate-limiting factor, even if it played a role in the process. Table 2 provides a summary of the fitting factors for different models.

**Table 2:** Parameters of the intraparticle diffusion model

Samples	Intraparticle diffusion model		
	*k*_D_ (mg g^−1^ h^−0.5^)	*C* (mg g^−1^)	*R* ^2^
1.0 g L^−1^	178.72	−64.216	0.9668
2.0 g L^−1^	335.35	−96.771	0.9661

That the adsorption equilibrium was established rapidly (within 20 min) is especially impressive, given the relatively low value of the BET surface area (65 m^2^ g^−1^). There are several possible reasons for this fast kinetic response: (1) the mesoporous nature of POP-AO (pore size 2.5–5 nm) provides for improved mass transfer and rapid diffusion of iodine molecules to the sites of chemical interaction, thus reducing the cost of internal mass transfer; (2) the high concentration of accessible amidoxime functional groups on the walls of the pores allows for an immediate chemisorption *via* Lewis acid–base bonds and eliminates the need for supplementary intraparticle mass transfer; and (3) because the electron-rich amidoxime groups possess a strong chemical attraction to the electrophilic nature of iodine molecules, the binding occurs quickly. The short time to reach equilibrium indicates the effectiveness of the chemical adsorption process and suggests that it is not limited by the physical mass transfer processes related to surface area. The opposite holds for physisorption; to obtain the same level of rate of adsorption, the surface area needs to be larger.

**Fig. 7:**
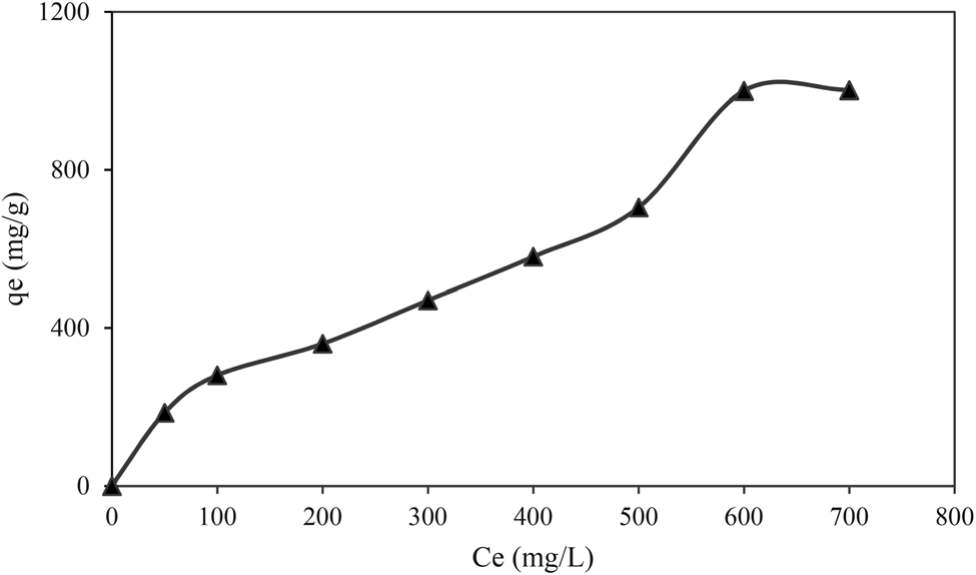
Effect of iodine concentration on the adsorption capacity of POP-AO.

The importance of the isothermal adsorption curve in creating precise equations that depict the adsorption process can help design adsorption systems.^36,37^ Using the isothermal adsorption curve during equilibrium makes it possible to accurately determine the adsorbent molecules among solid and liquid phases and the nonlinear relationship between the adsorbent and the pollutant in an isothermal adsorption process.^38^ The adsorption curve of COF-366-OA is shown in Fig. 7. Several isothermal adsorption equations, including Langmuir's and Freundlich's, were used in the investigation of the adsorption behavior of iodine on the POP-OA. Iodine adsorption behavior on the adsorbent was described by the Langmuir and the Freundlich isotherm models. The Langmuir model takes into consideration that the adsorption of the analyte is taking place in a monolayer on a homogeneous surface, implying that all adsorption sites are the same.^33^ In contrast, the Freundlich model is based on the premise that the adsorption process is conducted on multiple active sites on a heterogeneous surface and at a variety of intensities of adsorption. The following formulations of the Freundlich and Langmuir isotherm equations were used to assess the experimental data to identify the proper adsorption isotherm model:5
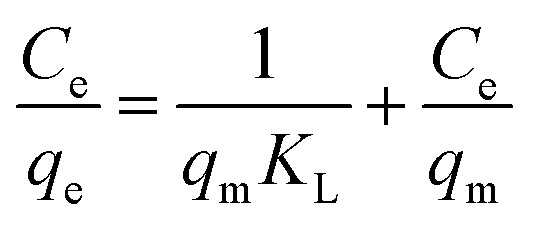

6 Ln *q*_e_ = Ln *K*_F_ + (1/*n*) Ln *C*_e_

The Langmuir model determined the maximum adsorption capacity (*q*_m_, mg g^−1^) to be 909.1 mg g^−1^. The constants associated with the Freundlich (*K*_F_) and Langmuir (*K*_L_) models were calculated as 0.973 mg g^−1^ and 0.005 L mg^−1^, respectively. Additionally, the Freundlich isotherm parameter (*n*) was 1.57. The experimental data were analyzed using both isotherm models to evaluate the nature of interactions between the adsorbent and adsorbate. The determination coefficients (*R*^2^) for the Langmuir and Freundlich models were determined to be 0.944 and 0.961, indicating a good fit for the adsorption behavior. Analysis revealed that the Freundlich model provided a better fit, as indicated by its higher determination coefficient than the Langmuir model. This suggests that iodine adsorption on POP-AO involves multilayer coverage and a heterogeneous distribution of active sites. Therefore, the adsorption process in aqueous solutions is characterized by spontaneity and randomness, which aligns well with the theoretical framework of the Freundlich isotherm.

Table 3 summarizes the maximum iodine adsorption capacities of various reported adsorbents and has been enhanced with their corresponding BET surface areas. This expanded comparison reveals that POP-AO achieves a superior and highly efficient performance. Notably, its high capacity (909.1 mg g^−1^) is coupled with excellent reusability (7 cycles) and rapid kinetics (20 min). When evaluated from multiple perspectives, POP-AO stands out: (1) efficiency: its exceptional capacity-to-surface-area ratio underscores that performance is driven by specific chemisorption from dense amidoxime sites rather than non-specific physisorption; (2) practicality: it maintains leading kinetics and cyclic stability; and (3) selectivity: its high capacity in the competitive liquid phase suggests strong affinity for iodine species. This combination of high efficiency per surface area, speed, and stability surpasses many advanced materials, including higher-surface-area COF composites and MOFs, under comparable liquid-phase conditions.

**Table 3:** Comparison of iodine *q*_m_ between POP-AO and other adsorbents

Adsorbent	*q*_m_ (mg g^−1^)	Reusability	Time (min h^−1^)	Surface area (m^2^ g^−1^)	Phase	Ref.
MIL-101(Cr)–SO_3_Ag	244.2	—	24 h	861	Liquid	11
MIL-101(Cr)–SO_3_H	94.1	—	24 h	1588	Liquid	11
Cotton fiber/COF monolith	823.9	7	20 min	166	Vapor	19
COF@cotton	533.0	5	5 h	124	Liquid	18
Fe_3_O_4_ (20%)@APC	844.6	6	40 min	—	Liquid	39
Thermal shock graphene	66.2	—	4 h	400	Vapor	40
POP-AO	909.1	7	20 min	65	Liquid	This work

The fact that the Freundlich model (*R*^2^ = 0.961) fitted better than Langmuir (*R*^2^ = 0.944) supports that POP-AO has a heterogeneous surface with different types of adsorption sites with varying binding energies. This heterogeneity can likely be associated with the presence of nitrogen and oxygen functionalities (amidoxime, pyrrole-N, and –NH groups) to capture iodine, where each functionality possibly contributes differently. Having a Freundlich parameter of *n* = 1.57 (where 1 < *n* < 10) indicates that the adsorption was favorable. This binding with multiple sites further is advantageous for retaining the high capacity through a wide concentration range, thus connecting the capacity/potential of POP-AO for treatment of iodine contaminated water, both in dilution and concentrated scenarios.

To optimize the desorption process for iodine recovery and identify the most effective eluent, systematic investigations were undertaken. Initially, comparisons of various types of eluents were made under identical conditions to determine desorption efficiency for NaCl, KCl, Na_2_SO_4_, and an organic solvent (ethanol or methanol), along with 0.1 mol per L dilute HCl, all held at 1.0 mol L^−1^ concentration of salt solution and desorbed for a period of 2 h at room temperature. Desorption efficiencies were found to be 95.8% NaCl, 94.9% KCl, 91.6% Na_2_SO_4_, 84.3% ethanol, 83.2% methanol, and 92.4% HCl. It may be concluded that, in general, monovalent salt solutions (NaCl and KCl) produce greater efficiencies regarding iodine desorption compared to organic solvents and divalent salts. Therefore, due to its effectiveness, low cost, benign nature to the environment, and availability, NaCl will be chosen as the eluent. Although 0.1 mol per L dilute HCl produced a high degree of desorption efficiency (92.4%), it was avoided as an eluting solvent due to the potential degradation of the amidoxime functionality that may occur during multiple applications of an acid. Optimization of the desorption process using NaCl was conducted by varying the concentration of NaCl solutions (0.5, 1.0, 1.5 mol L^−1^) and duration of desorption time (0.5, 1.0, 1.5, 2.0, 3.0 h) as indicated in Table S1. The results indicate that the optimum concentration of 1.0 mol per L NaCl, when held for an interval of 2 h, appears to provide the maximum desorption efficiency (95.8%) with little to no benefit of using a higher molarity or lengthening the time of desorption. The desorption mechanism contains disruption of Lewis acid–base interactions between I_2_ and amidoxime groups through ionic strength effects: (1) compression of the electrical double layer, and (2) competitive coordination of Na^+^/Cl^−^ ions. To avoid the possible blocking of the pores by salt accumulation, the adsorbent was washed with deionized water (3 × 10 mL) and ethanol (2 × 5 mL) after each cycle for the adsorbent regeneration. Desorption is primarily governed by ionic strength in saline solutions, which breaks the non-covalent interactions. Once the adsorbent loaded with iodine is subjected to a saline solution, the increase in ionic strength caused by the presence of NaCl reduces the stability of the charge-transfer complex formed between the electron-donating amidoxime groups and the electron-accepting I_2_ molecules. Besides this, the presence of solvated Na^+^ and Cl^−^ modifies the local solvation environment of the electron-donating sites of the amidoxime groups and the electron-accepting I_2_ molecules. In addition, the hydration shells of these dissolved ions compete at the interface, thus facilitating the transfer of molecular I_2_ from the adsorbent's surface into the aqueous solution. This helps ensure that the concentration gradient required for interfacial desorption is maintained. Importantly, this approach allows for the recovery of the molecular iodine while preserving the chemical integrity of the amidoxime groups, as demonstrated by the stable regeneration performance.

**Fig. 8:**
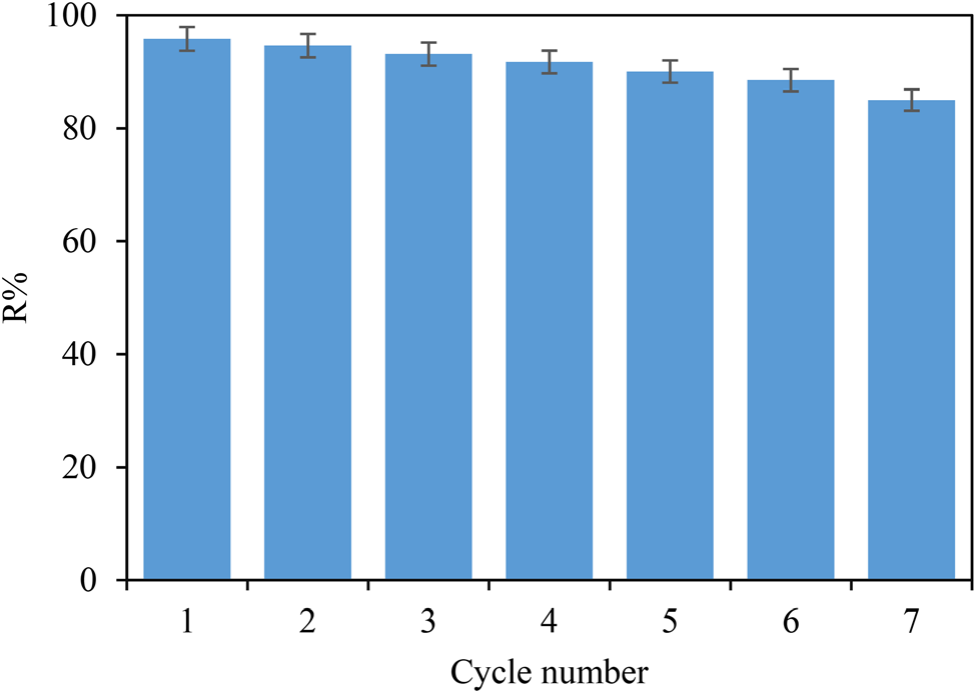
Effect of reusability for the obtained POP-AO on the iodine adsorption (RSD% = 2.2).

The reusability of POP-AO for iodine adsorption was evaluated through multiple cycles. In the initial cycle, iodine was adsorbed and then desorbed using a NaCl solution to remove the adsorbed iodine, preparing the material for subsequent cycles. Three adsorption and desorption cycles were carried out, resulting in seven cycles of reuse. It is illustrated in Fig. 8 that the iodine adsorption capacity of the adsorbent decreased by only 10.8% after seven cycles. These findings highlight the excellent durability and efficient reusability of synthesized POP-AO, making it a promising candidate for targeted iodine adsorption and broader environmental applications.

The small capacity loss of merely 10.8% after seven times point to the remarkable regenerability of POP-AO, which is fundamental for large-scale water treatment processes to be economically viable. This superb reusability is a result of the reversibility of Lewis acid–base interactions between the amidoxime groups and iodine; the NaCl solution is able to displace iodine that was adsorbed from the resin without destroying the functional groups. Retention of structural integrity as determined by FT-IR (Fig. S4) also ensures long-term operational stability by minimizing material replacement costs and secondary waste in remediation processes for radioactive iodine.

### 3.3. Adsorption mechanism

**Scheme 2:**
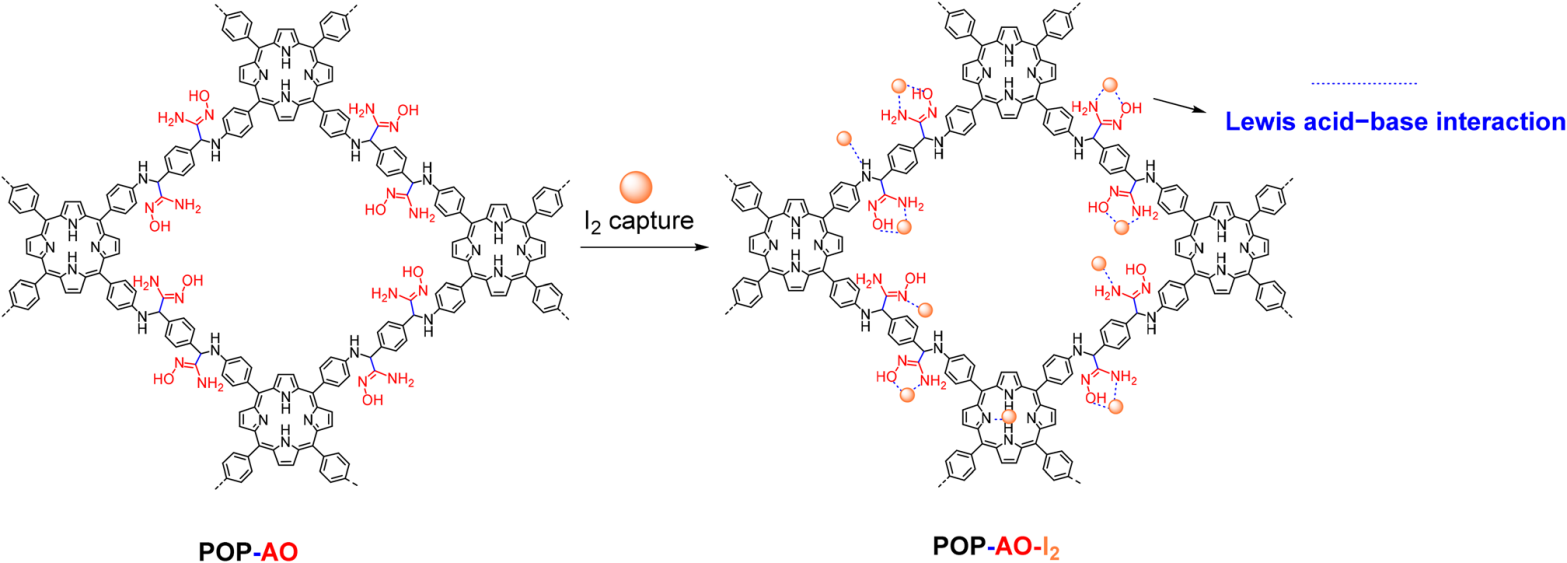
Illustration of the potential binding mechanism of iodine with POP-AO.

To investigate the adsorption mechanism, we compared the FTIR spectra of POP-AO before and after iodine uptake (Fig. S4). The spectrum of the iodine-loaded material (POP-AO–I_2_) shows significant peak broadening and distinct shifts, including the –NH/–OH stretch (from ∼3450 to 3399 cm^−1^) and the C=N stretch (from 1517 to 1514 cm^−1^). Furthermore, the appearance of new peaks at 1109, 1167, and 885 cm^−1^ indicates the formation of N,O–I_2_ complexes, confirming a chemical adsorption process. These changes confirm chemical changes in the POP-AO structure, particularly in the amidoxime groups (N/O functionalities), due to Lewis acid–base interactions.^12,14,24^ The observed peak shifts and the formation of new vibrational modes are characteristic of a charge-transfer mechanism, wherein lone-pair electrons from the N and O heteroatoms (particularly from the amide oxime group, with synergistic contributions from pyrrole N and –NH sites) are donated into the antibonding orbital (σ*) of I_2_. This electron transfer weakens the I–I bond and facilitates the formation of stable N,O–I_2_ complexes,^41,42^ a strategy effectively leveraged in POPs due to their tunable functionalization. The proposed mechanism for this interaction is illustrated in Scheme 2.

The Raman spectra of POP-AO after iodine adsorption (POP-AO–I_2_) were analyzed to investigate the nature of the adsorbed iodine species. The absence of distinct bands in the ∼110 cm^−1^ and ∼170 cm^−1^ regions,^43^ which are typically associated with polyiodide anions, indicates that iodine is not predominantly present in ionic or polyiodide forms. Instead, the Raman results suggest that iodine is mainly adsorbed in its neutral molecular form (I_2_).

From a practical standpoint, the cost-effectiveness and scalability of POP-AO, a member of the advanced POP family, must be evaluated with a balanced perspective. On one hand, its synthesis employs commercially available building blocks (though specialty precursors like porphyrin are not low-cost) and proceeds under moderately mild thermal conditions (70–120 °C). Furthermore, the successful scale-up of the pristine COF-366 scaffold itself by other researchers demonstrates a foundational pathway for production at larger volumes, which is promising for manufacturing the core framework of such materials. The efficient regeneration of POP-AO using low-cost NaCl solution and its high reusability further enhance its long-term economic profile for specific applications. On the other hand, the current multi-step synthesis, extended reaction times, and the cost of certain raw materials present significant challenges for immediate, low-cost mass production. These are common hurdles for this new generation of advanced functional materials, where initial focus is often on demonstrating exceptional performance such as the record-high iodine adsorption capacity (909.1 mg g^−1^) and selectivity demonstrated here. Therefore, POP-AO is positioned as a high-performance candidate for targeted, high-value applications like radioactive iodine capture, where performance can justify cost.

### 3.4. Analytical performance

To validate the analytical performance of the proposed approach for iodine determination, some key figures of merit have been investigated. Excellent linearity was obtained within the studied concentration range from 0.1 to 10 mg L^−1^ with *R*^2^ = 0.9984. Accordingly, the limit of detection calculated based on 3*S*_b_/*m* (where *S*_b_ is the standard deviation of the blank measurements and *m* represents the slope of calibration curve) was determined as 0.03 µg L^−1^, reflecting the capability of the method for trace-level analysis of iodine. The limit of quantification was calculated using LOQ = 10*S*_b_/*m* and was found to be 0.1 µg L^−1^. The precision of the method has been evaluated by repeatability studies and is expressed as RSD%. Analysis performed in five replicates at an iodine level of 0.5 mg L^−1^ provided an RSD value of 2.8%, confirming very good precision and reliability of the proposed procedure when routine iodine determination in aqueous samples is concerned.

### 3.5. Real samples

The effectiveness of the current method was evaluated by analyzing two real water samples, both spiked with iodine at concentrations of 0.3 and 0.5 mg L^−1^ respectively. One sample was tap water, and the other was well water, as shown in Table 4. Iodine was detected in the two water sample solutions. In confirming the accuracy of the current procedure, iodine was added to real water samples and analyzed using the method outlined in the current study. The data within this table demonstrated that the procedures used here would successfully extract iodine from real-world water samples.

**Table 4:** Determination of iodine in water samples

Sample	Iodine content (mg L^−1^)		*R*%
	Added	Found (± aRSD%)	
Tap water	—	—	—
	0.3	0.289 (±2.6)	96.3
	0.5	0.464 (±2.3)	92.8
Well water	—	—	—
	0.3	0.274 (±2.7)	91.3
	0.5	0.453 (±3.1)	90.6

a Relative standard deviation.

## 4. Conclusion

An amidoxime-functionalized swelling polymer (POP-AO) was successfully synthesized through post-synthetic modification of COF-366 and used as an effective adsorbent for removing iodine from water solutions. The adsorption tests showed an optimal pH of 6 and a quick equilibrium time of 20 minutes, indicating fast adsorption. Impressively, POP-AO had an iodine uptake capacity of 909.1 mg g^−1^ at 298 K, exceeding many other reported adsorbents. Additionally, the material maintained high adsorption efficiency even after seven regeneration cycles, demonstrating its strong reusability. Mechanistic studies confirmed that amidoxime groups mainly capture iodine by Lewis acid–base coordination, forming charge-transfer complexes. This process involves electron donation from the N and O atoms to the antibonding orbital of I_2_. This work emphasizes the potential of functionalized POPs and opens the door for their use in wider environmental cleanup efforts.

## Conflicts of interest

There are no conflicts to declare.

## Supplementary Material

RA-016-D5RA09537A-s001

## Data Availability

Data supporting the findings of this study can be obtained from the corresponding author upon reasonable request. Supplementary information (SI): 1 – instruments. 2 – Fig. S1. Comparative FT-IR spectra: COF-366, COF-366-CN, and POP-AO. 3 – Fig. S2. SEM images of COF-366 at different magnifications. 4 – Fig. S3. XRD pattern of COF-366. 5 – Fig. S4. Nitrogen adsorption–desorption isotherm for POP-AO; the corresponding pore size distribution plot is shown in the inset. 6 – Fig. S5. TGA profile for POP-AO, showing mass loss as a function of temperature. 7 – Fig. S6. FT-IR spectra of POP-AO (top, blue) and POP-AO-I_2_ (bottom, red). 8 – Fig. S7. The effect of time on the *q*_*t*_*.* 9 – Fig. S8. Raman spectra of POP-AO after iodine adsorption. 10 – Table S1. Optimization of desorption conditions for iodine from adsorbent. See DOI: https://doi.org/10.1039/d5ra09537a.
