# Supplementary material for: Efficient iodine capture from water using a functionalized covalent organic framework
Source: RSC Adv. 2026 Feb 19;16(11):10129–39. doi: 10.1039/d5ra09537a (PMC12919391; doi:10.1039/d5ra09537a)
Supplement: RA-016-D5RA09537A-s001 [file RA-016-D5RA09537A-s001.pdf]

Supplementary Information (SI) for RSC Advances.
This journal is © The Royal Society of Chemistry 2026

Supporting Information

Efficient Iodine Capture from Water Using a Functionalized Covalent Organic Framework

Mahsa Jahantigh,[1] Mostafa Khajeh,[1,*] Ali Reza Oveisi,[2,*] Saba Daliran,[2] and Mansoureh Rakhshanipour[1]

[1] Department of Chemistry, Faculty of Sciences, University of Zabol, P.O. Box: 98615-538, Zabol, Iran

[2] Department of Organic Chemistry, Faculty of Chemistry, Lorestan University, Khorramabad 68151-44316, Iran.

Corresponding authors:

E-mail: m_khajeh@uoz.ac.ir (M. Khajeh) and oveisi.a@lu.ac.ir (A. R. Oveisi), Fax: +98-543-2226765

Instruments

PerkinElmer Spectrum spectrometer (Version 10.01.00) with KBr pellet preparation was used to acquire Fourier-transform infrared (FT-IR) spectra. Diffraction analysis was carried out using a Philips X'Pert diffractometer (Cu Kα radiation, λ = 1.5406 Å). To determine the elemental composition of the samples, energy-dispersive X-ray spectroscopy (EDX) was employed using a TESCAN MIRA3 field-emission scanning electron microscope (FE-SEM). UV-Vis absorption measurements were conducted on an Agilent 8453 spectrophotometer at λ = 458 nm to monitor iodine concentration in the supernatant solution over time. The thermal stability of the samples was evaluated by thermogravimetric analysis (TGA) using a Mettler Toledo TGA/DSC instrument. Measurements were conducted from ambient temperature to 700 °C under air constant heating rate of 10 °C/min. Nitrogen physisorption measurements were performed at 77 K using a Micromeritics TriStar II Plus surface area and porosity analyzer. Prior to analysis, the samples were degassed under vacuum at 120 °C for 12 hours. Raman spectra were collected using an RM2000 confocal Raman spectrometer.

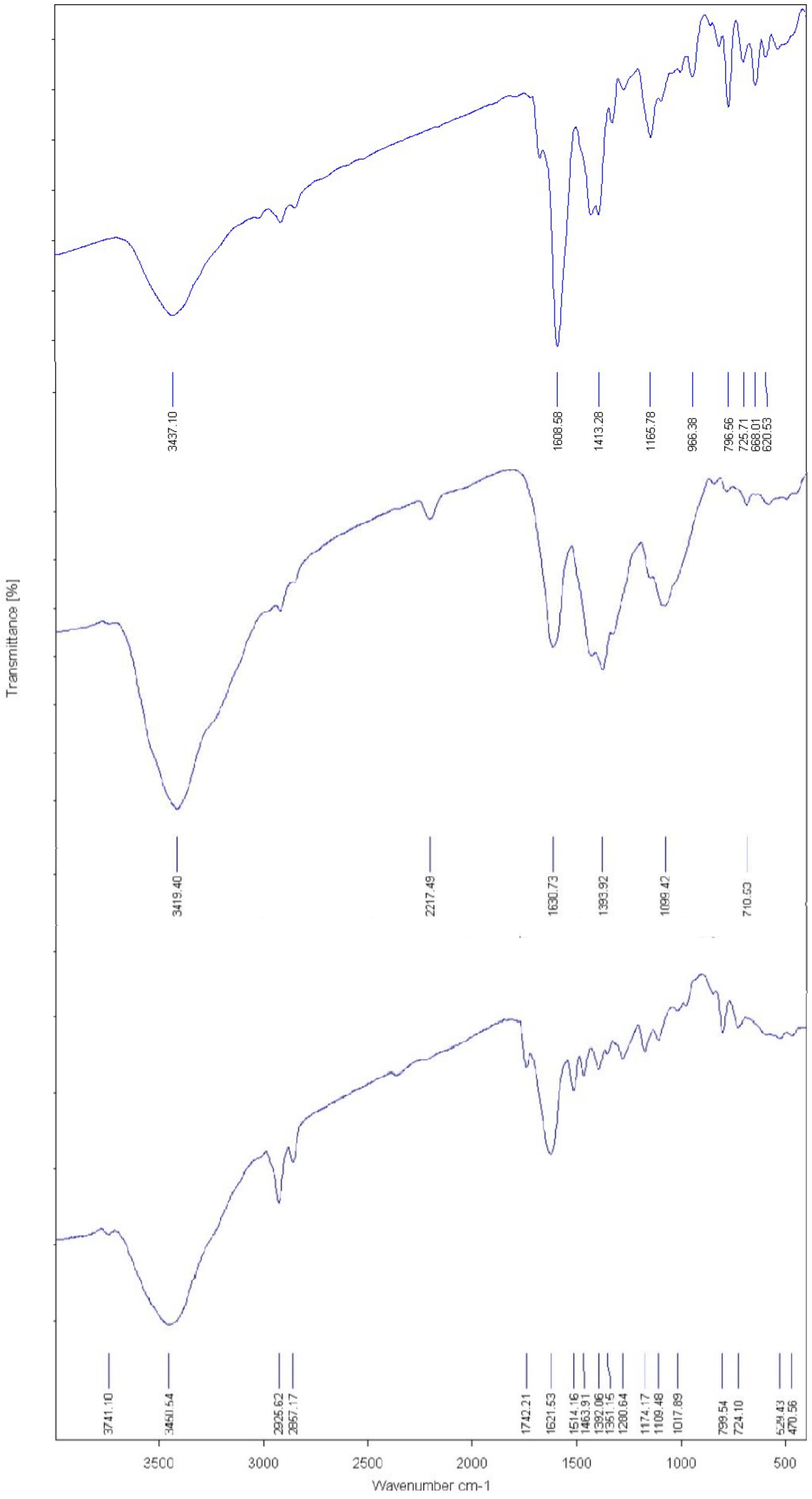

Transmittance [%]
Wavenumber cm-1
3500
3000
2500
2000
1500
1000
500
3437.10
1608.58
1413.28
1165.78
966.38
796.56
725.71
668.01
620.53
3419.40
2217.49
1630.73
1393.92
1099.42
710.53
3741.10
3450.54
2925.62
2857.17
1742.21
1621.53
1514.16
1463.91
1392.06
1351.15
1280.64
1174.17
1109.48
1017.89
799.54
724.10
529.43
470.56

Figure S1. Comparative FT-IR spectra: COF-366 (top), COF-366-CN (middle), and POP-AO (bottom).

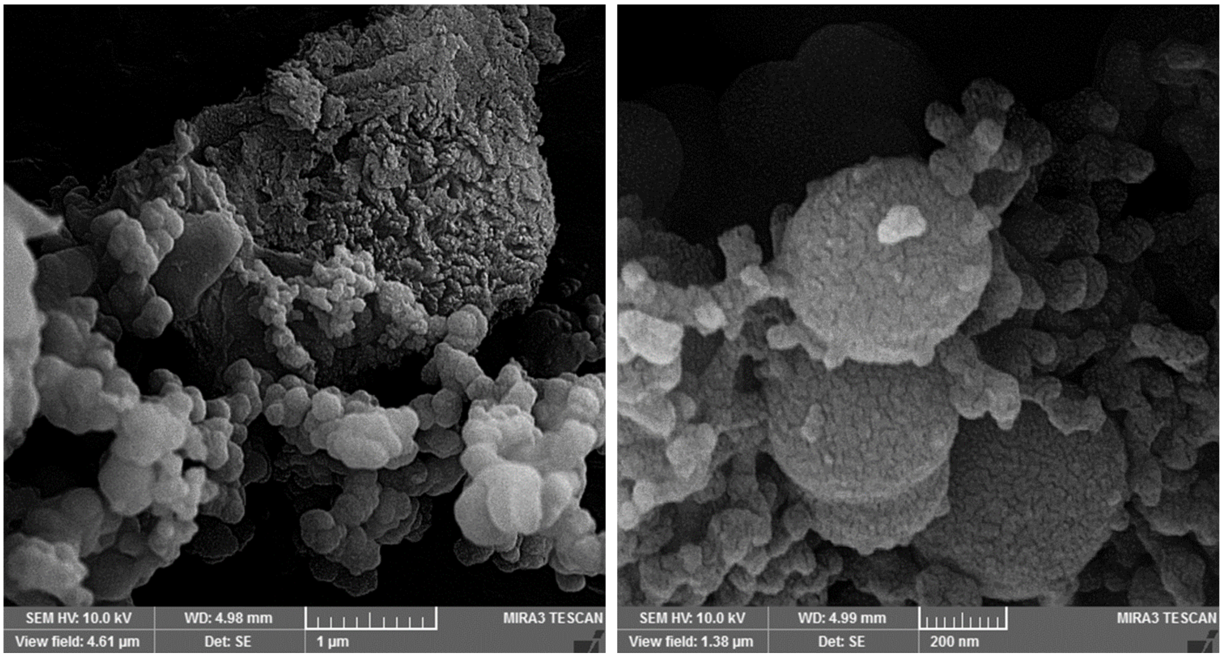

Figure S2. SEM images of COF-366 at different magnifications.

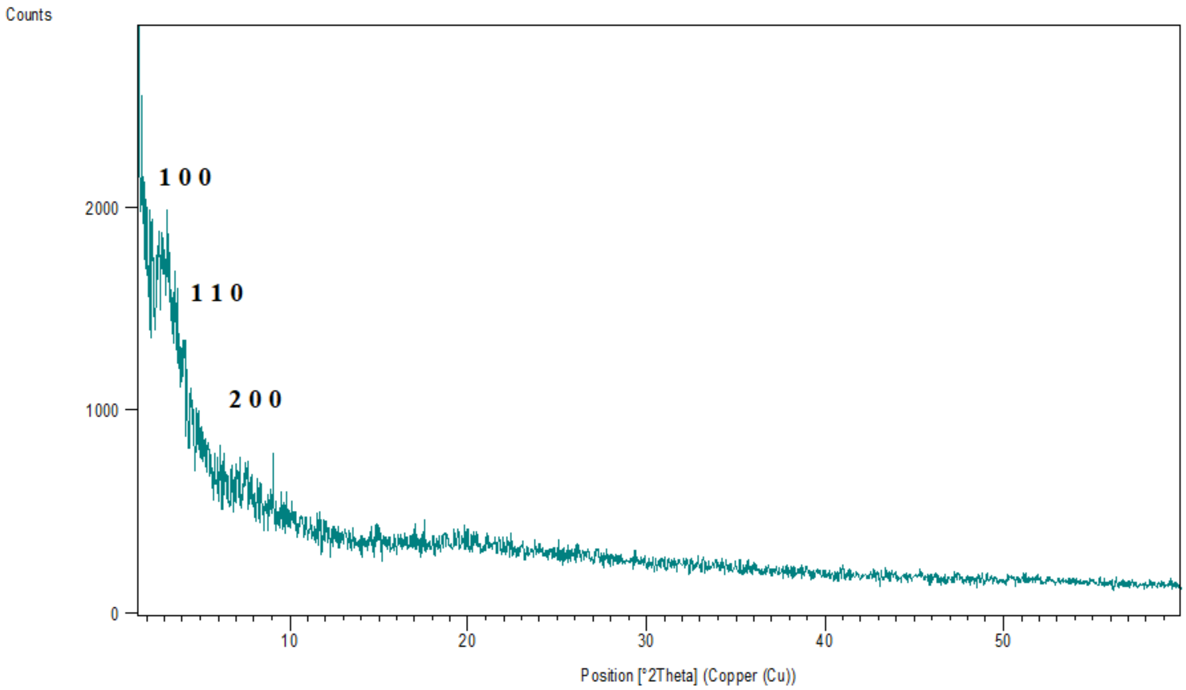

Figure S3. XRD pattern of COF-366.

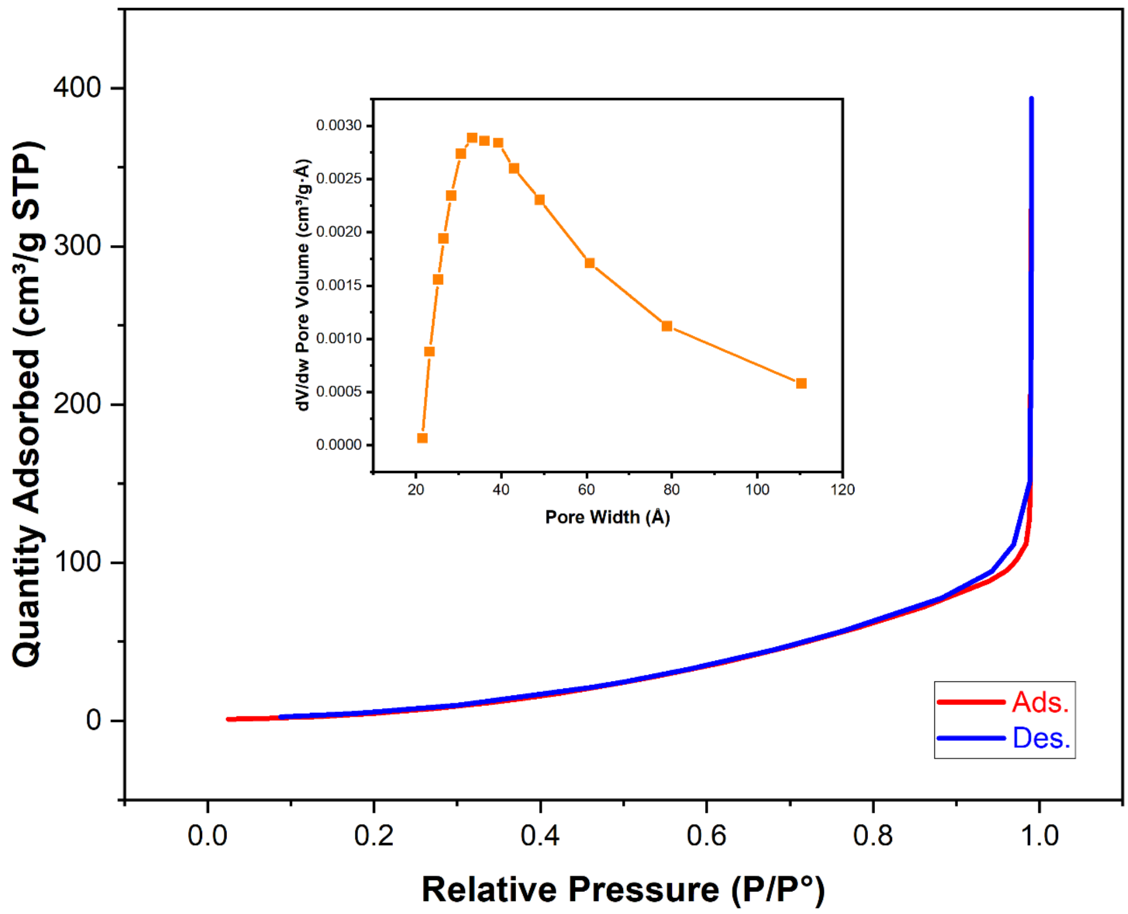

Figure S4. Nitrogen adsorption-desorption isotherm for POP-AO; the corresponding pore size distribution plot is shown in the inset.

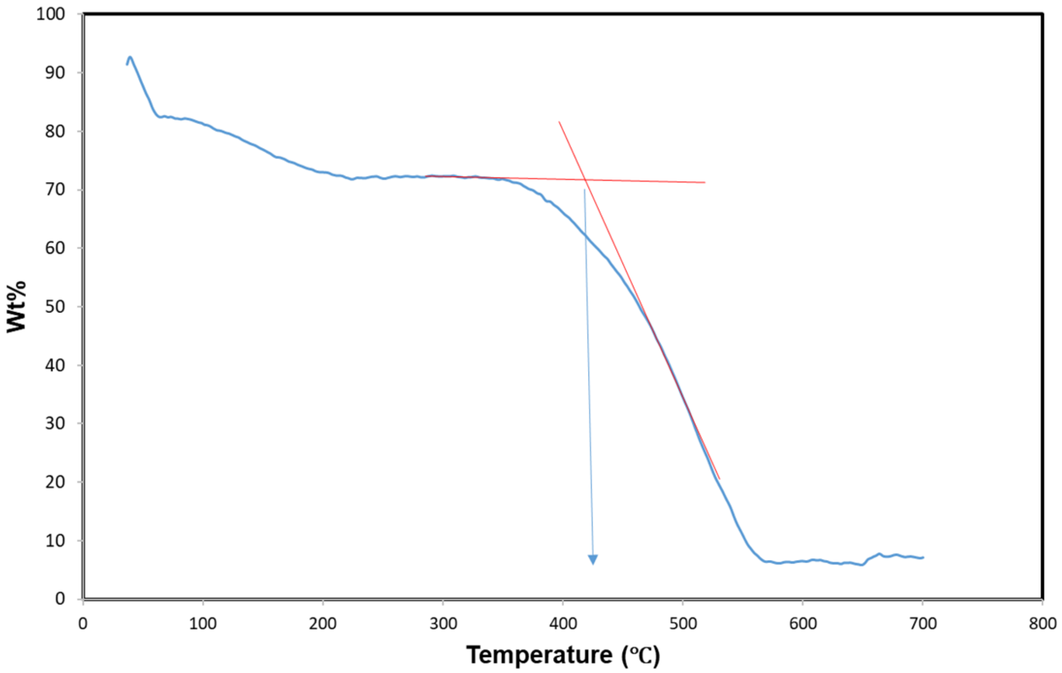

Figure S5. TGA profile for POP-AO, showing mass loss as a function of temperature.

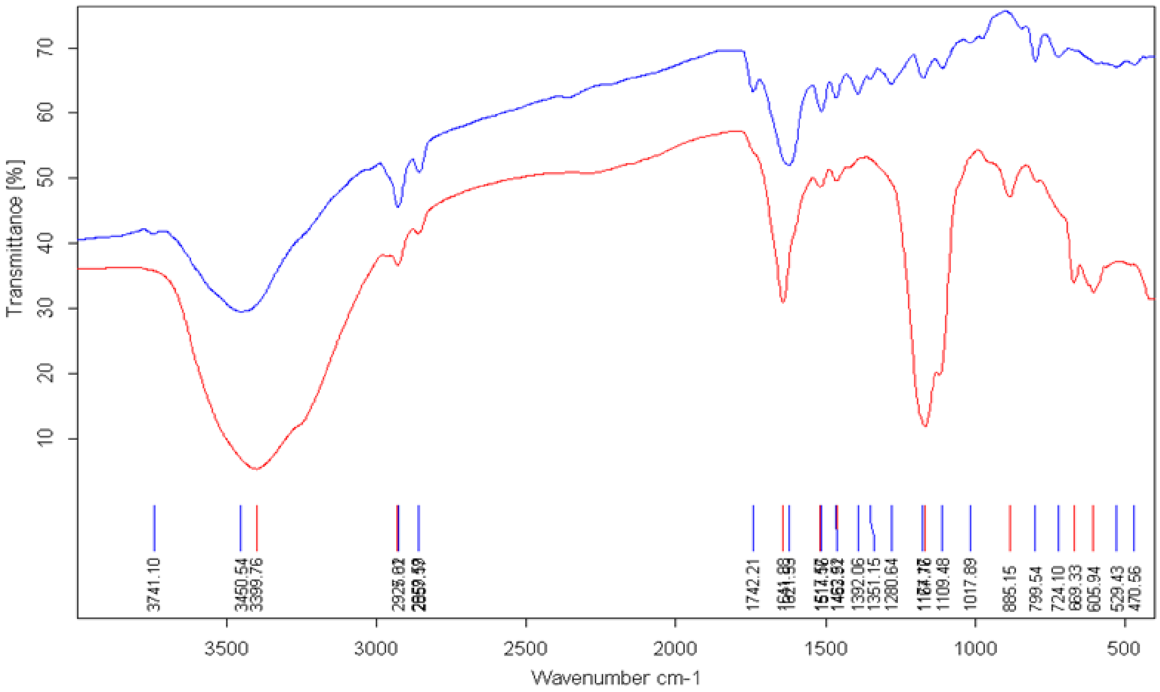

Figure S6. FT-IR spectra of POP-AO (top, blue) and POP-AO-I_2 (bottom, red).

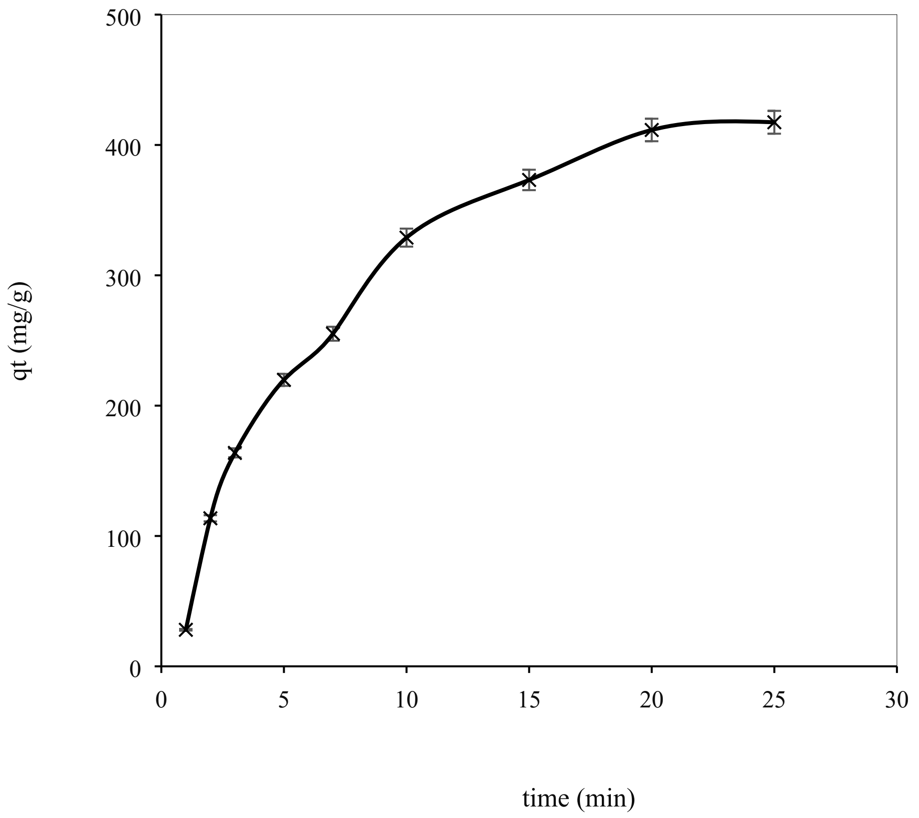

Figure S7. The effect of time on the q_t (RSD% =2.1)

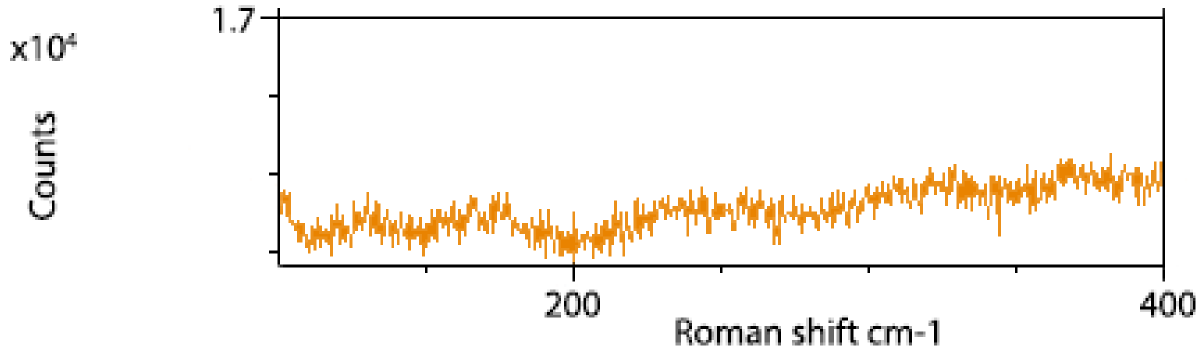

Figure S8. Raman spectra of POP-AO after iodine adsorption.

Table S1. Optimization of desorption conditions for iodine from adsorbent.

NaCl Concentration (mol/L)	Desorption time (h)	Desorption Efficiency (%)
0.5	0.5	69.3
0.5	1.0	76.5
0.5	1.5	84.2
0.5	2.0	88.6
0.5	3.0	89.1
1.0	0.5	81.4
1.0	1.0	90.3
1.0	1.5	94.1
1.0	2.0	95.8
1.0	3.0	95.9
1.5	0.5	84.1
1.5	1.0	91.6
1.5	1.5	94.5
1.5	2.0	95.8
1.5	3.0	96.1
